# Electrochemical Performance of a PVDF-HFP-LiClO_4_-Li_6.4_La_3.0_Zr_1.4_Ta_0.6_O_12_ Composite Solid Electrolyte at Different Temperatures

**DOI:** 10.3390/nano12193390

**Published:** 2022-09-28

**Authors:** Xinghua Liang, Yujuan Ning, Linxiao Lan, Guanhua Yang, Minghua Li, Shufang Tang, Jianling Huang

**Affiliations:** 1 Guangxi Key Laboratory of Automobile Components and Vehicle Technology, Guangxi University of Science and Technology, Liuzhou 545006, China; 2 School of Electrical Technology, Guangdong Mechanical & Electrical Polytechnic, Guangzhou 510515, China; 3 College of Automotive Engineering, Liuzhou Institute of Technology, Liuzhou 545616, China

**Keywords:** lithium-ion battery, solid-state electrolyte, composite, electrochemical performance

## Abstract

The stability and wide temperature performance range of solid electrolytes are the keys to the development of high-energy density all-solid-state lithium-ion batteries. In this work, a PVDF-HFP-LiClO_4_-Li_6.4_La_3_Zr_1.4_Ta_0.6_O_12_ (LLZTO) composite solid electrolyte was prepared using the solution pouring method. The PVDF-HFP-LiClO_4_-LLZTO composite solid electrolyte shows excellent electrochemical performance in the temperature range of 30 to 60 °C. By assembling this electrolyte into the battery, the LiFePO_4_/PVDF-HFP-LiClO_4_-LLZTO/Li battery shows outstanding electrochemical performance in the temperature range of 30 to 60 °C. The ionic conductivity of the composite electrolyte membrane at 30 °C and 60 °C is 5.5 × 10^−5^ S cm^−1^ and 1.0 × 10^−5^ S cm^−1^, respectively. At a current density of 0.2 C, the LiFePO_4_/PVDF-HFP-LiClO_4_-LLZTO/Li battery shows a high initial specific discharge capacity of 133.3 and 167.2 mAh g^−1^ at 30 °C and 60 °C, respectively. After 50 cycles, the reversible electrochemical capacity of the battery is 121.5 and 154.6 mAh g^−1^ at 30 °C and 60 °C; the corresponding capacity retention rates are 91.2% and 92.5%, respectively. Therefore, this work provides an effective strategy for the design and preparation of solid-state lithium-ion batteries.

## 1. Introduction

Lithium-ion batteries (LIBs) exhibit high energy density, a low self-discharge rate, and a long cycle life, and they have been widely used in various portable electronic devices [1,2,3]. LIBs continue to prove their irreplaceable value as power suppliers in a variety of applications. However, liquid LIBs include potential explosion hazards due to their flammability; thus, its security issues remain unresolved. Solid-state electrolytes have attracted a wide range of research interests because they are highly flame-retardant and have good flexibility. For example, polymer electrolytes [4,5,6,7,8,9,10], ceramic electrolytes [11,12,13,14,15,16,17], and ceramic/polymer composite electrolytes [18,19,20,21,22,23] have been intensively investigated in recent years.

Among these, ceramic/polymer composite electrolytes have attracted wide attention due to their high ionic conductivity, elastic stiffness, high shear modulus, high flexibility, and lower density, which can effectively inhibit the growth of lithium dendrite and make better contact with the electrode interface, thus improving the electrochemical performance of solid-state lithium-ion batteries. Zhang et al. [24] prepared a PVDF/LLZTO composite electrolyte by dispersing LLZTO ceramic powder into a polymer matrix. The ionic conductivity of the composite electrolyte is 5 × 10^−4^ S cm^−1^ at room temperature, and it exhibits high mechanical strength and good thermal stability. In the LiCoO_2_/Li battery composed of the composite electrolyte, the initial specific discharge capacity reaches 150 mAh g^−1^, and the capacity retention rate reaches 98% after 120 cycles at 0.4 C. Gu et al. [25] reported a PVDF-HFP/LLZTO composite solid electrolyte membrane (CSE) with a simple synthesis route and complete raw materials. The mixed electrolyte contains a wide electrochemical stability window (~5 V), excellent mechanical properties (tensile strength over 13 MPa, young’s modulus > 50 MPa), good ionic conductivity (3.2 × 10^−4^ S cm^−1^) at room temperature, and good electrochemical properties (about 150 mAh g^−1^ at 0.1 C; the coulomb efficiency is 99% after 50 cycles). Although good electrochemical performance was achieved in composite solid electrolytes, novel solid electrolytes with better electrochemical performance need to be developed to match the requirements for a practical battery.

Generally, the electrochemical performance of these solid electrolytes is measured at a certain temperature. As an important performance for practical application, the electrochemical performances of the solid electrolytes at wide temperature ranges were rarely reported. However, the applied temperature has an obvious influence on the electrochemical performance. In this work, the PVDF-HFP-LiClO_4_-Li_6.4_La_3_Zr_1.4_Ta_0.6_O_12_ (LLZTO) composite solid electrolyte was prepared using the solution pouring method, and its electrochemical performance was investigated systematically in the temperature range of 30 to 60 °C. It is found that the PVDF-HFP-LiClO_4_-LLZTO composite solid electrolyte shows excellent electrochemical performance in the temperature range of 30 to 60 °C. By assembling this electrolyte into the battery, the LiFePO_4_/PVDF-HFP-LiClO_4_-LLZTO/Li battery shows outstanding electrochemical performance at 30 and 60 °C. It is noted that the electrolyte and the assembled battery show better electrochemical performance at 60 °C than at 30 °C.

## 2. Experimental Section

### 2.1. Preparation of PVDF-HFP-LiClO_4_-LLZTO Composite Solid Electrolyte

**Figure 1:**
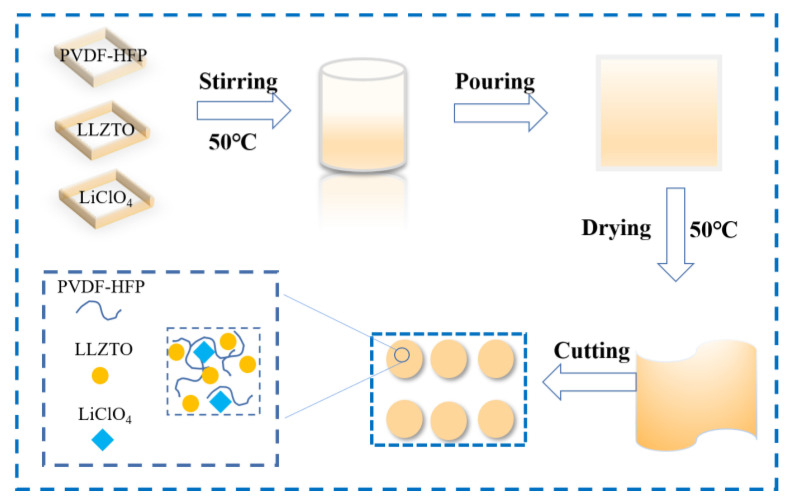
Schematic pattern of the preparation process of CSE.

CSE was prepared using the solution casting method, and the preparation process is shown in Figure 1. Typically, 4 g of PVDF-HFP (Mn = 600,000, Aladdin, Shanghai, China) and a certain amount of LiClO_4_ (99%, Aladdin, Shanghai, China) were dissolved/dispersed in N-N dimethylformamide (DMF, 99.8%, Aladdin, Shanghai, China). LLZTO (99%, Kocrystal, Shenzhen, China) was introduced under moderate stirring, and the temperature was set at 50 °C. Then the slurry was transferred into the PTFE mold for static flow casting. PVDF-HFP-LiClO_4_-LLZTO CSE can be exfoliated after vacuum-drying at 60 °C for 12 h. Finally, the electrolyte membrane was cut into small discs with a diameter of 18 mm and stored in a glove box (<0.01 PPM H_2_O and O_2_).

### 2.2. Physical Characterization

The phase analysis of the materials was measured on an X-ray diffractometer (XRD, DX-2700, Dandong, China, Cu-Kα, 40 kV × 40 mA). The surface structure of the solid electrolyte films was characterized by scanning electron microscopy (SEM, Phenom Pharos G2, Shanghai, China) equipped with energy dispersive spectrometry (EDS). Raman spectra were collected by a Raman microscope (ATR8000, Fujian, China). Thermogravimetric analysis (TGA) was conducted using a TG analysis system (NetzschF3Tarsus, Bayern, Germany) from 30 to 800 °C with a heating rate of 10 °C min^−1^.

### 2.3. Electrochemical Tests

High ionic conductivity at operating temperature is one of the prerequisites for the application of CSE in solid-state batteries. The electrochemical impedance spectroscopy (EIS) of CSE was measured on an electrochemical workstation (DH 7000, Donghua, Jiangsu, China) using button-mounted symmetrical cells assembled from stainless steel (SS)/CSE/SS. The EIS was conducted from 10^6^ Hz to 0.01 Hz with an amplitude of 10 mV. The ionic conductivity σ was calculated according to the following formula [24]:

(1) $$\sigma =\frac{L}{\mathrm{RS}}$$

where σ is the ionic conductivity (S/cm), L is the thickness of the film (cm), R is the local minimal resistance over all the impedance spectrum (Ω), and S is the contact area between the film and the SS (cm^2^).

The electrochemical stability window of CSE was determined by linear sweep voltammetry (LSV) using an SS/CSE/Li semi-symmetric cell with a scanning rate of 5 mV S^−1^ in the voltage range from 2.5 V to 6.0 V. The lithium-ion migration number (T_Li_^+^) of the CSE at 30 °C and 60 °C was measured by the combined measurement of EIS and DC polarization on the same electrochemical station using a symmetric Li/CSE/Li battery. T_Li_^+^ is calculated according to the following formula [26,27]:

(2) $$T_{{\mathrm{Li}}^{+}}=\frac{I_{\mathrm{SS}}\left(\Delta V-I_{0}R_{0}\right)}{I_{0}\left(\Delta V-I_{\mathrm{SS}}R_{\mathrm{SS}}\right)}$$

where T_Li^+^_ represents the number of lithium-ion migrations in the electrolyte, I_0_ and I_SS_ are the current values at the beginning and after the DC polarization is stabilized, R_0_ and R_SS_ are the impedance values before and after the DC polarization, and ΔV is the voltage applied to both ends of the battery at 50 mV.

Electrochemical measurements of the cells were carried out by a battery test system (Neware, Dongguan, China). Commercial LiFePO_4_ (LFP, Macklin, Shanghai, China) is used as the cathode active material without further modification, and a lithium plate is used as the anode. The cathode is made of a paste of 80 wt.% LFP, 10 wt.% PVDF, and 10 wt.% conductive carbon black cast on aluminum foil. The active material loading of the prepared cathode is about 1.8 mg cm^−2^. The assembled LFP/CSE/Li cells were tested in the voltage range of 2.8 to 4.0 V. Liquid electrolyte (1.0 M LiPF_6_ ethyl carbonate (EC), diethyl carbonate (DEC) and diethyl carbonate (DMC) (V:V:V = 4:3:3)) was added to the surface of the electrolyte membrane to improve the interface contact between the membrane and the electrode.

## 3. Results and Discussion

**Figure 2:**
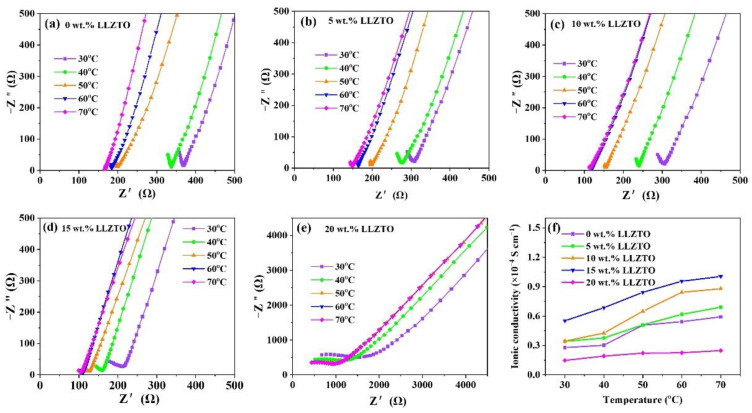
(**a**–**e**) EIS and (**f**) corresponding calculated ionic conductivity of CSE with different LLZTO content at different temperatures.

The addition of LLZTO inorganic filler can effectively reduce the crystallinity of the polymer, introducing a large number of Li^+^ migration channels, thus improving the ionic conductivity of the electrolyte membrane and enhancing its mechanical properties [28]. However, excessive LLZTO makes it difficult to completely peel the electrolyte membrane from the mold, and the ionic conductivity of the electrolyte membrane decreases due to the agglomeration of the LLZTO filler. Considering the influence of LLZTO on the ionic conductivity of the electrolyte membrane, the CES were prepared with different amounts of LLZTO (0~20 wt.%), namely 0 wt.% LLZTO, 5 wt.% LLZTO, 10 wt.% LLZTO, 15 wt.% LLZTO, and 20 wt.% LLZTO, respectively. Figure 2a–e shows the EIS of CSE with different LLZTO content at different temperatures; corresponding calculated ionic conductivity of the electrolyte membranes is shown in Figure 2f. It is found that the ionic conductivity of CES increases with the increase in LLZTO content at first, then shows a rapid decline after the LLZTO content is more than 15 wt.%. Therefore, the CSE with 15 wt.% LLZTO was used as the studied object for the following discussion. Obviously, the resistance of the CSE decreases with the increase in temperature, and the calculated ionic conductivity of CSE at 30 °C, 40 °C, 50 °C, 60 °C, and 70 °C are 5.5 × 10^−5^, 6.9 × 10^−5^, 8.4 × 10^−5^, 9.6 × 10^−5^, and 1.0 × 10^−4^ S cm^−1^, respectively. It should be pointed out that there is little difference between 60 °C and 70 °C, so 60 °C is chosen as the upper-temperature limit for the following discussion.

**Figure 3:**
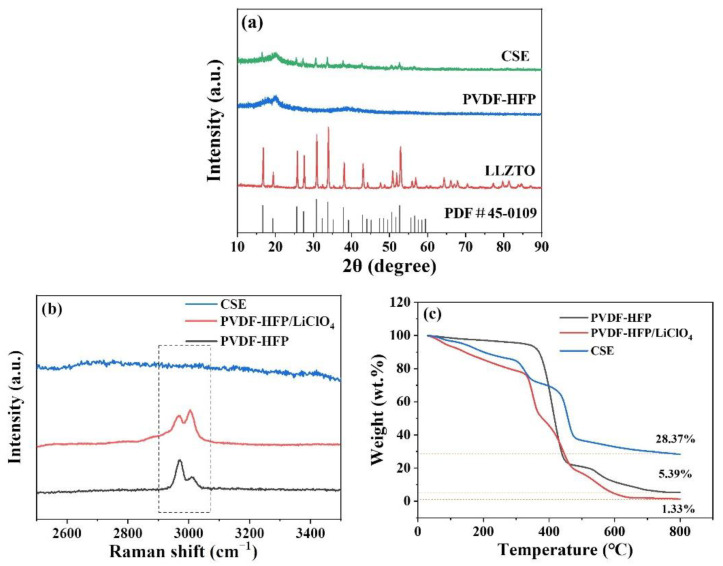
(**a**) XRD, (**b**) Raman, and (**c**) TG of PVDF-HFP, PVDF-HFP-LiClO_4_ and CSE.

Figure 3a shows the XRD patterns of the samples. For LLZTO powder, the diffraction peaks are matched with cubic garnet Li_5_La_3_Nb_2_O_12_ (PDF#45-0109), indicating that LLZTO is a typical cubic phase. With respect to CSE, the diffraction peak of LLZTO can also be observed. Figure 3b shows the Raman curves of the materials; it can be seen that the PVDF-HFP peak appeared in the PVDF-HFP/LiClO_4_ membrane, while there was none in the CSE, indicating that PVDF-HFP reacted with LLZTO. Thermal stability is an important indicator of composite polymer electrolytes. Figure 3c shows the TG analysis of the materials; the test temperature ranges from 30 °C to 800 °C. It can be seen that the weight loss temperature of PVDF-HFP is about 450 °C, PVDF-HFP/LiClO_4_ starts to decompose at 350 °C, and CSE loses weight at 456 °C. The weight loss of the PVDF-HFP, PVDF-HFP/LiClO_4_ and CSE at 800 °C are 98.67%, 94.61%, and 71.63%, respectively, indicating that the weight loss of CSE decreases after the addition of LLZTO, which is beneficial to improve the thermal stability of CSE.

**Figure 4:**
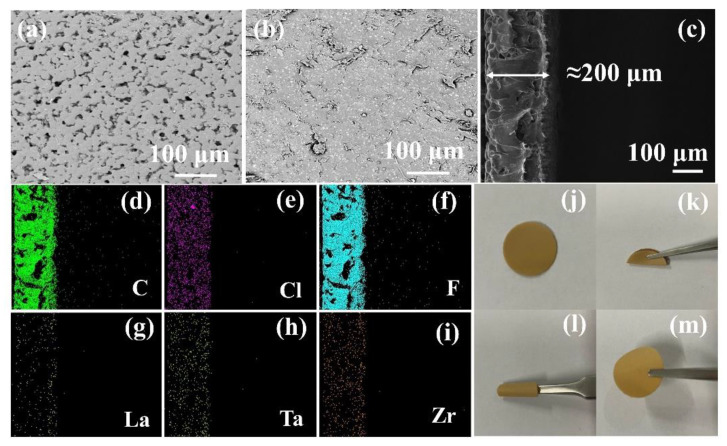
(**a**,**b**) SEM images and (**c**) cross-sectional SEM images of CSE, (**d**–**i**) corresponding EDS mapping of CSE and (**j**–**m**) macro picture of CSE in different physical states.

The morphology of the CSE was characterized by a scanning electron microscope (SEM). Figure 4a–c shows SEM images of the front, back, and cross-section of the CSE sample, respectively. The positive surface of the electrolyte film has a thickness of about 200 μm, and there are some holes, which may be due to the uneven volatilization of the solvent. Some solid particles and roughness can be observed on the back of the electrolyte film, which may be caused by part of ceramic oxide LLZTO sinking into the bottom of the mold during the drying process of the slurry. The roughness on the back of the electrolyte film is caused by the surface roughness of the PTFE mold. It can be seen from the cross-section that the ceramic oxide LLZTO filler is evenly distributed in the CSE. It is also explained that this heterostructure contributes to the high performance of all-solid-state lithium batteries (ASSLB), as smooth surfaces in contact with the cathode (polymer-rich) will help to reduce interface resistance due to their soft properties, while rough surfaces in contact with the lithium anode (LLZTO-rich) will be beneficial. The EDS mapping of C, Cl, F, La, Ta, and Zr are shown in Figure 4d–i. It can be observed that the LLZTO ceramic particles are uniformly dispersed in the electrolyte membrane. Figure 4j–m shows photos of the electrolyte membranes in different physical states; obviously, the prepared CSE shows excellent mechanical flexibility and can be bent and wound. The excellent mechanical flexibility should be beneficial to inhibit the formation of lithium dendrite.

**Figure 5:**
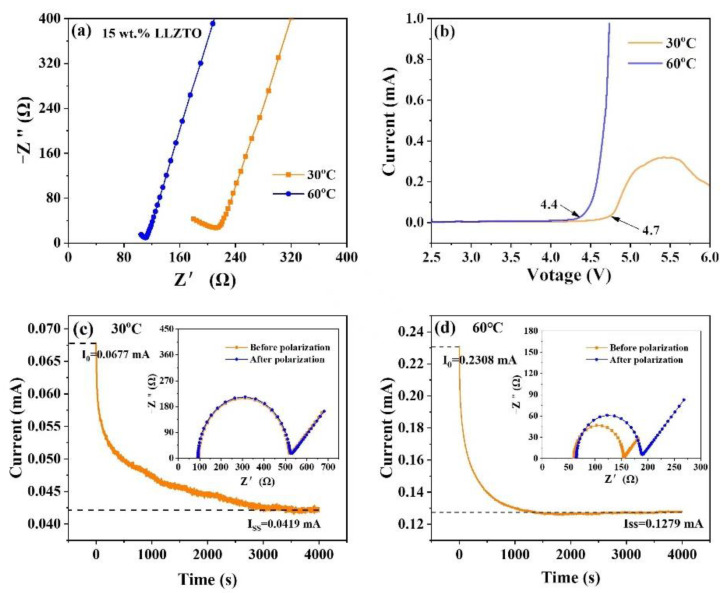
(**a**) Impedance diagram, (**b**) LSV diagram, and (**c**,**d**) DC polarization and AC impedance curves of CSE at 30 and 60 °C.

As mentioned above, the ionic conductivity of CSE at 60 °C is 1.0 × 10^−4^ S cm^−1^, which is higher than that at 30 °C, being 5.5 × 10^−5^ S cm^−1^; the corresponding EIS is shown in Figure 5a,b shows the electrochemical stability windows at the temperatures of 30 °C and 60 °C. The results show that the electrochemical stability window of the CSE at 60 °C is 4.4 V, while the electrochemical stability window at 30 °C is 4.7 V. Li^+^ migration number is an important parameter to evaluate ion mobility [29]; Figure 5c,d shows the polarization curves and initial/steady impedance curves of the electrolyte membrane at 30 °C and 60 °C. The T_Li_^+^ of the composite solid electrolyte at 60 °C is 0.32, higher than that that at 30 °C (0.21). The addition of LLZTO powder as an inorganic filler not only improves the ionic conductivity of the material, but also has a positive impact on the electrochemical window and the migration number of lithium ions [30]. Moreover, the addition of LLZTO ceramic fillers has been reported to slack the polymer chain. Therefore, the interaction between the inorganic filler and the polymer chain promotes the movement of the chain segments and accelerates the dynamic process between them [31].

**Figure 6:**
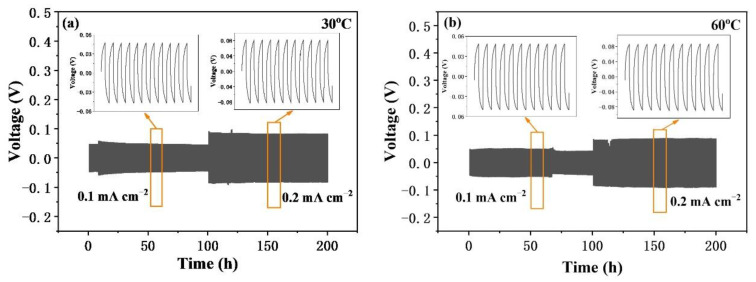
Constant current charge and discharge cycling of Li/CSE/Li symmetric batteries at (**a**) 30 °C and (**b**) 60 °C.

In order to explore the effect of CSE on the stability of lithium metal and the transport capacity of lithium ions, a symmetric battery composed of a lithium metal anode and a lithium metal cathode was charged and discharged at 0.5 h in a constant current. Figure 6a,b shows the process of lithium plating/stripping of symmetric cells at different temperatures. After 100 h of cycling at 0.1 mA cm^−2^ and 0.2 mA cm^−2^, the batteries exhibit stable lithium plating/stripping processes at all given current densities, and the composite solid electrolyte battery exhibits very stable polarization voltage in the 200 h cycle, at either 30 °C or 60 °C. This result shows that the CSE can achieve reversible electroplating and stripping of lithium metal without obvious lithium dendrites.

**Figure 7:**
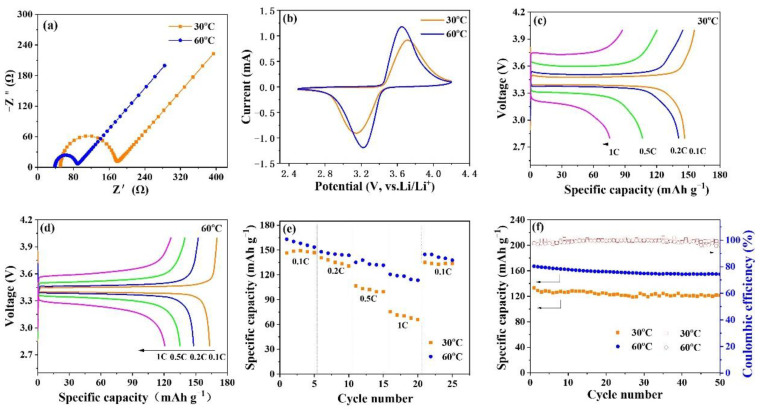
(**a**) Impedance diagram, (**b**) CV curves, (**c**,**d**) initial charge–discharge curves, (**e**) rate capability and (**f**) cycling life of LFP/CSE/Li battery at 30 and 60 °C.

To explore the charge transfer resistance of the battery at different temperatures, EIS measurement of the half-cell assembled by CSE, cathode LFP, and anode Li at different temperatures was conducted, as shown in Figure 7a. Each EIS spectrum consists of a semicircle, caused by charge transfer resistance (Rct), and a diagonal line corresponding to the diffusion of Li^+^ [32]. It can be seen that the semicircle at 60 °C is smaller than that at 30 °C, indicating that the charge transfer resistance of the battery decreases with the increase in temperature. Figure 7b shows the cyclic voltammetry (CV) of CSE at different temperatures between 2.5 V and 4 V, at a scanning rate of 0.5 mV s^−1^. In the CV curves shown in Figure 7b, a remarkable reduction peak appears at ~3.0 V for all the cells, and a strong oxidation peak appears at ~3.8 V, corresponding to the lithium ion dilithium and stripping from the LFP. When observed carefully, it can be seen that the CV curve for 60 °C has a larger redox peak area, indicating that it has a larger specific capacity [33].

To verify the applicability of the prepared electrolyte membranes at different temperatures, the LFP/CSE/Li battery was assembled, and its electrochemical performances were measured at 30 °C and 60 °C. Figure 7c,d shows the initial charge and discharge (CD) curves of the assembled ASSLB, with different rates at different temperatures, in the voltage range of 2.8~4.0 V. The initial CD curve presents a typically smooth and monotonous voltage plateau due to the extraction/insertion of Li^+^ from the LFP active substance. The voltage platform gap of the battery at 30 °C is larger than that at 60 °C, indicating that there is a bigger polarization in the charging and discharging process at 30 °C. In addition, for all applied current densities, the battery shows a larger charge-discharge capacity at 60 °C than that at 30 °C, fitting well with the results of the CV.

It is noteworthy that the rate performance of the assembled lithium-ion battery is closely related to the ionic conductivity of the prepared CSE. The CSE has higher ionic conductivity at 60 °C than at 30 °C, so the battery assembled using this membrane shows better rate performance at 60 °C, as shown in Figure 7e. At a current density of 0.1 C, the assembled battery has a specific discharge capacity of 147.9 mAh g^−1^ and 158.2 mAh g^−1^ at 30 °C and 60 °C, respectively. After experiencing the cycle of 0.2, 0.5, and 1 C and recovering to 0.1 C, the specific discharge capacity of the battery can be restored to 133.9 mAh g^−1^ and 141.8 mAh g^−1^, respectively, indicating that the battery assembled by this composite solid electrolyte has good reversible performance.

Figure 7f shows the cycling life of the LFP/CSE/Li battery with 0.2 C at 30 °C and 60 °C. It can be seen that the initial specific discharge capacities at 30 °C and 60 °C are 133.3 and 167.2 mAh g^−1^, respectively. After 50 cycles, the specific discharge capacities are 121.5 and 154.6 mAh g^−1^, respectively; the corresponding retention rates are 91.2 and 92.5%, respectively. The results show that the battery has better cycling life at 60 °C because the high temperature accelerates the relative motion of Li^+^ and promotes the migration of Li^+^. In short, the results of electrochemical measurements obtained above indicate that the prepared CSE enables the solid-state battery to show good rate capability, high reversible capacity, and good cycling life at 30 °C and 60 °C.

## 4. Conclusions

The PVDF-HFP-LiClO_4_-LLZTO composite solid electrolyte was prepared using the solution pouring method, and its electrochemical performance was investigated systematically in the temperature range of 30 to 60 °C. The ionic conductivity of the composite electrolyte membrane at 30 °C and 60 °C is 5.5 × 10^−5^ S cm^−1^ and 1.0 × 10^−4^ S cm^−1^, respectively, and the electrochemical stability window of the composite electrolyte membrane at 30 °C and 60 °C is 4.7 V and 4.4 V, respectively. By assembling this electrolyte into the battery, the LiFePO_4_/PVDF-HFP-LiClO_4_-LLZTO/Li battery shows outstanding electrochemical properties at the temperatures of 30 and 60 °C. At a current density of 0.2 C, the LiFePO_4_/PVDF-HFP-LiClO_4_-LLZTO/Li battery shows a high initial specific discharge capacity of 133.3 and 167.2 mAh g^−1^, respectively, at 30 °C and 60 °C. After 50 cycles, the reversible electrochemical capacity of the battery at 30 °C and 60 °C is 121.5 and 154.6 mAh g^−1^, respectively, and the corresponding capacity retention rates are 91.2% and 92.5%, respectively. In addition, the battery shows excellent rate capability, especially at 60 °C. We believe this work stimulates further interest in solid composite electrolytes for high-energy density lithium batteries.

## Data Availability

Not applicable.
